# Microstructures and Corrosion Properties of Wire Arc Additive Manufactured Copper–Nickel Alloys

**DOI:** 10.3390/ma17040876

**Published:** 2024-02-14

**Authors:** Jie Song, Xavier A. Jimenez, Albert C. To, Yao Fu

**Affiliations:** 1 Department of Aerospace and Ocean Engineering, Virginia Tech, Blacksburg, VA 24061, USA; jiesong@vt.edu; 2 Department of Materials Science and Engineering, Virginia Tech, Blacksburg, VA 24061, USA; 3 Department of Mechanical Engineering & Materials Science, University of Pittsburgh, Pittsburgh, PA 15261, USA; xaj5@pitt.edu (X.A.J.); albertto@pitt.edu (A.C.T.)

**Keywords:** 70/30 copper–nickel, wire arc additive manufacturing, dendritic feature, chemical segregation, corrosion properties, sodium chloride solution

## Abstract

The 70/30 copper–nickel alloy is used mainly in critical parts with more demanding conditions in marine settings. There is a need for innovative methods that offer fast production and cost-effectiveness in order to supplement current copper–nickel alloy manufacturing processes. In this study, we employ wire arc additive manufacturing (WAAM) to fabricate the 70/30 copper–nickel alloy. The as-built microstructure is characterized by columnar grains with prominent dendrites and chemical segregation in the inter-dendritic area. The aspect ratio of the columnar grain increases with increasing travel speed (TS) at the same wire feed speed (WFS). This is in contrast with the equiaxed grain structure, with a more random orientation, of the conventional sample. The sample built with a WFS of 8 m/min, TS of 1000 mm/min, and a track distance of 3.85 mm exhibits superior corrosion properties in the 3.5 wt% NaCl solution when compared with the conventional sample, as evidenced by a higher film resistance and breakdown potential, along with a lower passive current density of the WAAM sample. The corrosion morphology reveals the critical roles played by the nickel element that is unevenly distributed between the dendrite core and inter-dendritic area.

## 1. Introduction

Copper–nickel (Cu–Ni) alloys find widespread application in marine settings, including heat exchangers, piping, and valves. Their resistance to corrosion and bio-fouling makes them the preferred choice for highly corrosive environments [1,2,3,4], such as seawater, particularly when exceptional thermal conductivity is essential [5,6]. The 70/30 Cu–Ni alloy is mainly used in critical parts working under more demanding conditions. Cu–Ni alloys primarily consist of α-phase-based single solid solutions due to the mutual solubility of Cu and Ni [7]. Traditional manufacturing processes for Cu–Ni alloys involve casting and forging, which are time-consuming and expensive, especially when fabricating parts with intricate designs.

Consequently, there is a need for innovative methods that offer fast production and cost-effectiveness. Therefore, there has been a notable surge in the exploration of additive manufacturing (AM) methods, such as powder bed fusion, and direct energy deposition (DED) [8,9,10,11]. One significant drawback associated with metal-powder-based AM techniques is their relatively low deposition rate, typically ranging from 0.12 to 0.6 kg per hour. In contrast, wire arc DED or wire arc additive manufacturing (WAAM), a high-deposition-rate technique that utilizes modified welding equipment, stands out for its low capital investment requirements and the ability to achieve deposition rates up to a hundred times higher. Consequently, it has emerged as a subject of immediate interest within the scientific and research community [12,13]. While several welding modes are suitable for WAAM, the cold metal transfer (CMT) method is favored due to its ability to lower heat input and enhance arc stability through the physical retraction of the wire, facilitating droplet detachment [14].

However, the existing body of literature on additive manufacturing, in particular WAAM, using Cu–Ni raw materials is very limited [15,16]. In this study, 70/30 Cu–Ni alloys are printed by WAAM using the CMT method. The microstructures and defects of the as-built sample have been characterized as a variation of printing conditions. The corrosion properties have been investigated employing electrochemical approaches in sodium chloride solution.

## 2. Materials and Methods

### 2.1. Process Parameter Development

The machine used in this study is the Arc605 machine, manufactured by Gefertec (Berlin, Germany), a 5-axis CNC machine utilizing a Fronius TPS 400i power source from Wels, Austria. A 1.2 mm diameter 70/30 Cu–Ni (TECHALLOY^®^ 413) wire from Lincoln Electric (Cleveland, OH, USA) was employed for printing on a 70/30 Cu–Ni plate (C71500) supplied by Marmetal Industries (Hatboro, PA, USA). The plate is in wrought state and has also been investigated for its microstructures and corrosion properties and compared with its WAAM counterparts. The chemical concentration of the wire and the plate are provided in Table 1. A CMT power source from Fronius was utilized, and the shielding gas used was a mixture of 75% Ar and 25% He from Linde, flowing at a rate of 15 L per minute. The wire feed speed (WFS) was kept as 8 m/min, and the torch speed (TS) varied between 500 and 1000 mm/min. The interpass temperature in the WAAM process is the temperature at which the next deposition layer operates. Maintaining the interpass temperature within a suitable range is crucial when influencing microstructures and mechanical properties. There have been studies on a variety of different alloys related to the effect of interpass temperature in WAAM on grain size, phase constitution, and mechanical properties [17,18,19,20,21]. In our work, a pyrometer Metis M318 was used to keep the interpass temperature of the walls below 200 °C.

**Figure 1:**
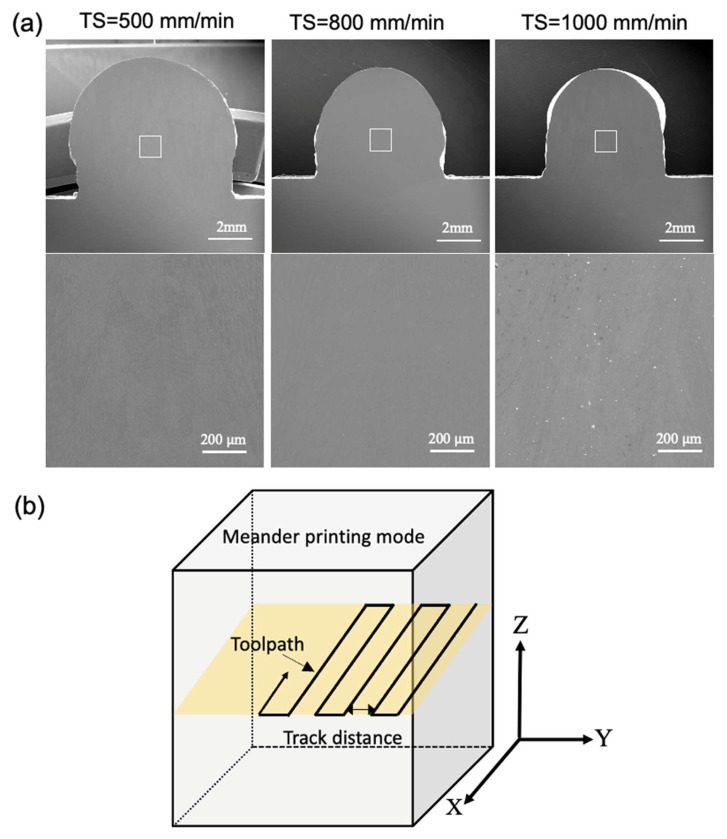
(**a**) SEM image (based on secondary electron diffraction) of (**upper panel**) cross-section of the single-bead 3-layer build and (**lower panel**) magnified SEM image of selected area in the upper panel (indicated by the white square); the three columns correspond to the TSs equal to 500, 800, and 1000 mm/min. (**b**) Schematic of the deposition strategy.

Initially, a single-bead wall, 100 mm in length, was printed for 3 layers in order to screen the processing parameters (i.e., WFS and TS) in the medium-to-high production domain and in turn to ensure a high-quality build free of waviness and with other types of defects kept to a minimum by scanning electron microscopy (Figure 1a). Subsequently, the block was printed using a meander deposition path (Figure 1b) based on the parameters that produced successful single bead walls, as listed in Table 2, with variations in the track distance. The determination of track distance is as follows. The single-bead wall at different TS has a different width (Figure 1a). At TS = 500, 800, and 1000 mm/min, the single-track widths were estimated as 7.5 mm, 6 mm, and 5.5 mm, respectively. Then, the track distance was chosen as approximately 40% and 70% of the single-track width, thus resulting in different choices at different TSs, as shown in Table 2. The choice of track distance leads to different overlaps between adjacent scanning tracks, and allowed us to understand how this affects the resultant microstructures and corrosion performance.

### 2.2. Microstructural Characterization, Corrosion and Mechanical Tests

The microstructures of the samples were assessed using optical microscopy (OM), scanning electron microscopy (SEM), energy-dispersive X-ray spectroscopy (EDS) and electron backscatter diffraction (EBSD). To prepare the samples for OM/SEM/EBSD analysis, they underwent mounting, grinding, and either vibration polishing or electropolishing. Electropolishing was carried out using a solution consisting of 12.5% sulfuric acid and 87.5% methanol at a potential of 25 V and at room temperature. Etching was performed electrolytically in a 10% saturated oxalic acid solution at 12 V for a brief period.

For electrochemical testing, the specimens were EDM machined from the build block in the size of 10 × 10 × 5 mm with the broad surface along the XY and YZ build plane. The surface and bottom area were avoided during the harvest of specimens. The samples were sealed with epoxy resin, exposing an area of 0.32 cm^2^ and ground to a P2400 grit finish. A three-electrode cell was used with a graphite counter electrode and the aqueous Ag/AgCl reference electrode as the reference point for all potentials. The test solution was 3.5 wt.% (0.61 M) NaCl, and the experiments were conducted at room temperature under natural aeration. The sample was immersed in the solution under an open circuit condition for 3 h prior to the electrochemical impedance spectroscopy (EIS) and cyclic polarization (CP) tests. The samples after each electrochemical test (either EIS or CP) were ground to expose fresh surfaces. The EIS test was performed using a 10 mV (rms) perturbation from 100 kHz to 10 mHz. Fitting was performed with Gamry software (version 7.9.0). Cyclic polarization curves follow the forward scan rate of 0.1667 mV/s, starting from −250 mV (vs. open circuit potential). Reversal occurs when the current density reaches 3 mA/cm^2^ at a reverse scan rate of 1.667 mV/s. Electrochemical measurements are recorded using a Gamry potentiostat/galvanostat. The reported data represent the average of at least three measurements.

## 3. Results

### 3.1. Microstructural Analysis

**Figure 2:**
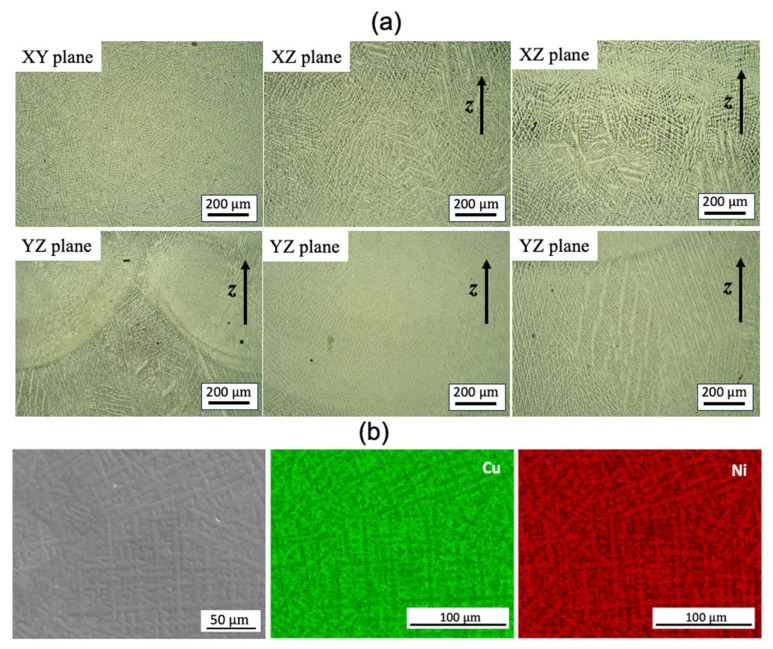
(**a**) OM images of the dendrite features on different build planes and (**b**) SEM image and EDS map scanning of WAAM sample 1.

The microstructure examined by optical microscopy (OM) analysis revealed no porosity or macro/microscopic cracks (Figure 2a). Dendrites were clearly observed, with the primary dendrite arm spacing (PDAS) varying in different locations, particularly adjacent to the fusion lines/melt pool boundaries. The dendrites mostly grew perpendicular to the melt pool boundaries (Figure 2a). According to Hunt’s model [22], the PDAS increases as the temperature gradient decreases. The heterogeneous PDAS indicates the presence of a complicated solidification environment. The energy-dispersive X-ray spectroscopy (EDS) analysis of the selected domain confirmed the presence of solute segregation, with Ni elements preferentially locating in the dendrite cores, when compared with the inter-dendritic spacing (Figure 2b). This aligns with the expectations based on the phase diagram of the Cu–Ni binary alloy. The formation of intermetallic compounds was not found, as Cu and Ni are completely soluble in each other as a single-phase solid solution.

**Figure 3:**
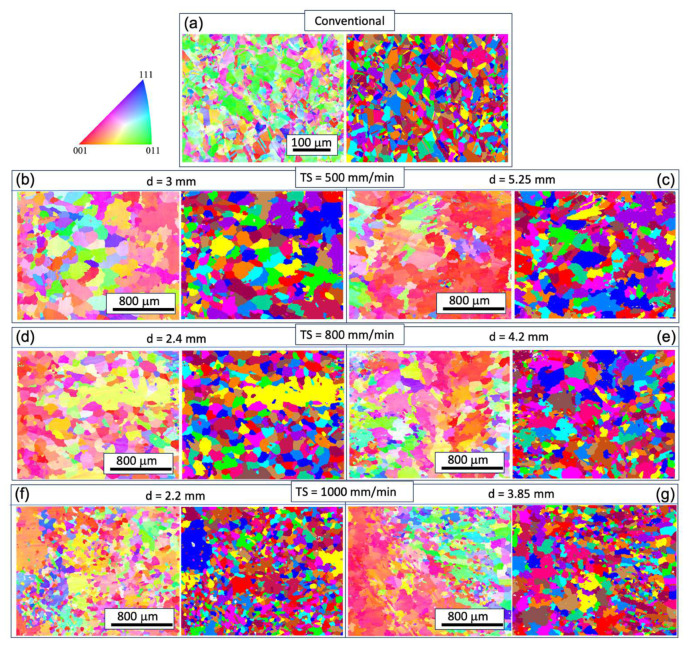
IPF maps for grain orientation and grain structure maps on the XY plane at different track distances (d) with respective torch speed (TS) as follows: TS = 500 mm/min, 800 mm/min, and 1000 mm/min; subfigure (**a**) corresponds to the conventional sample, and subfigures (**b**–**g**) correspond to WAAM samples 1–6.

**Figure 4:**
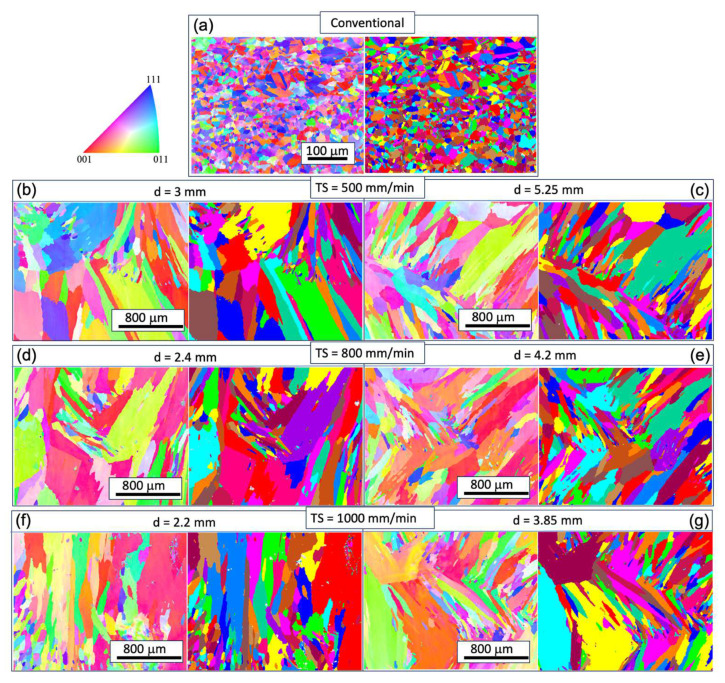
IPF maps for grain orientation and grain structure maps on the YZ plane at different track distances (d) with respective torch speed (TS) as follows: TS = 500 mm/min, 800 mm/min, and 1000 mm/min; subfigure (**a**) corresponds to the conventional sample, and subfigures (**b**–**g**) correspond to WAAM samples 1–6.

To understand the microstructural and texture changes that occur during the printing process, EBSD analyses were conducted for all of the WAAM samples. For comparison, the analysis of the conventional sample is also presented. In Figure 3 and Figure 4, inverse pole figure (IPF) maps on the XY plane (perpendicular to the build direction) and YZ plane (along the build direction) are provided. The XY cross-sections of the grains exhibit an equiaxed shape, while the YZ plane cross-sections display a columnar shape. The longitudinal directions of the grains tend to deviate from the build direction (z-axis). Given that the layer thickness is estimated to be 2~3 mm (Figure 1), the grains likely do not span multiple layers, in contrast with the observations in Inconel 718 [23]. This phenomenon is potentially related to the heat flux direction and temperature gradient within the melt pool, which is worthy of further analysis. Epitaxial growth from previous grains occurs at the melt pool boundary, but these grains do not seem to be in preferential crystal growth direction and are thus mostly hindered in the growth process, as seen in Figure 4.

**Figure 5:**
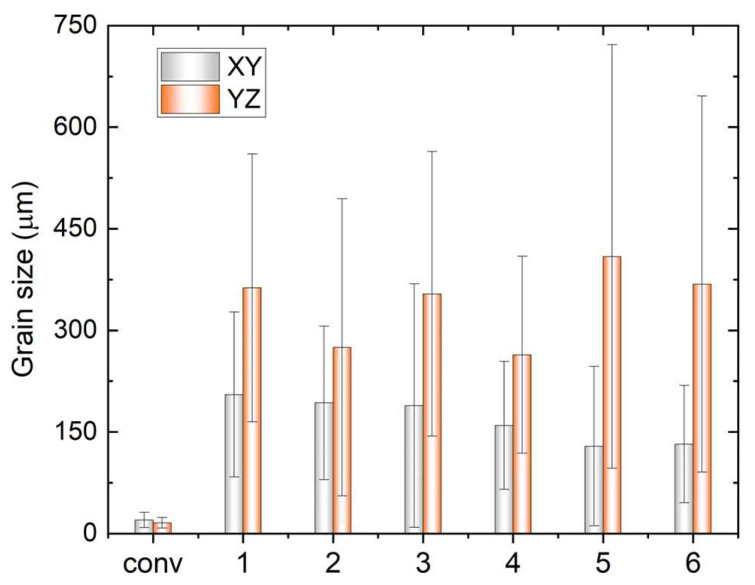
Variation of grain size in the XY and YZ planes of the conventional sample (conv) and WAAM samples under different printing conditions; x-axis labels 1–6 indicate the WAAM samples 1–6.

The grain size analysis in Figure 5 reveals an increased aspect ratio of the columnar grains with rising TS, indicating smaller sizes in the XY plane and larger sizes in the YZ plane. Additionally, the size on the YZ plane decreases with increasing track distance at the same WFS and TS, attributed to a faster cooling rate that benefits grain refinement. It is noteworthy that the very small grains in Figure 3 and Figure 4 may result from the intersection of some dendrite arms with the examined planes.

**Figure 6:**
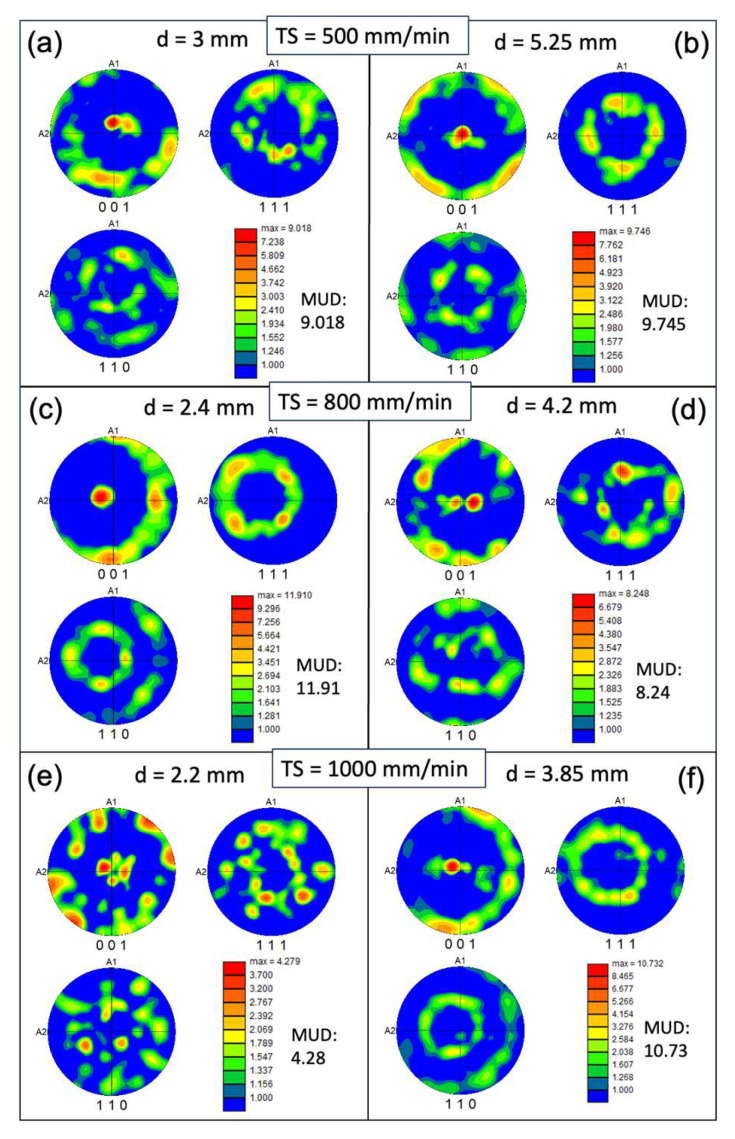
Pole figure (PF) colored orientation maps measured on the XY plane of the WAAM samples at different track distances (d) with respective torch speed (TS) as follows: TS = 500 mm/min, 800 mm/min, and 1000 mm/min; subfigures (**a**–**f**) correspond to WAAM samples 1–6.

In contrast with the conventional condition, the inverse pole figure (IPF) map indicates a concentration of the XY plane in the 〈001〉 texture (Figure 3). However, approximately 30–40% of the area in the XY cross-section consists of randomly oriented grains. The pole figure (PF) plots (Figure 6) further illustrate the strength of the texture for the three families of planes in face-centered cubic (FCC) on 〈001〉, 〈110〉, and 〈111〉. These plots confirm that the 〈001〉 texture component is the strongest among the three. The maximum multiple of uniform density (MUD) value is highest for sample 3 (MUD = 11.9), even though that of 〈001〉 is not strongly aligned along the z direction. Conversely, the MUD value is lowest for sample 5 (MUD = 4.3), indicating a weak texture.

**Figure 7:**
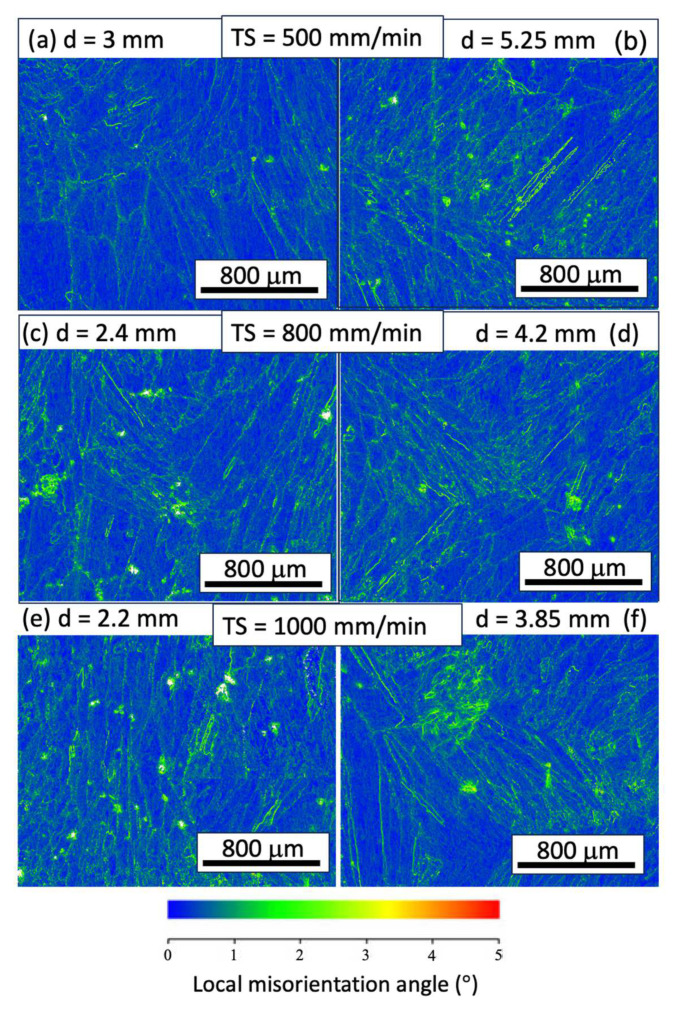
Kernel average misorientation (KAM) maps of WAAM samples at different travel speeds (TS) of the YZ plane with respective TS as follows: TS = 500 mm/min, 800 mm/min, and 1000 mm/min; subfigures (**a**–**f**) correspond to WAAM samples 1–6.

Figure 7 illustrates the kernel average misorientation (KAM) and grain boundaries of the EBSD analysis, presented for the YZ planes. The KAM provides a qualitative description of the degree of homogenization of plastic deformation or the density of defects such as dislocations, lamellar dislocations, and sub-grain boundaries. Higher KAM values indicate a greater degree of plastic deformation or a higher density of defects. The KAM analysis of the three samples reveals a low degree of dislocation in most regions, suggesting that residual stresses in the additively fabricated samples have been adequately released, and that deformation within the grains is minimal. This phenomenon can be attributed to the relatively slower cooling rate in the WAAM process compared with other powder-based AM processes. Additionally, the stress-relieving annealing effect caused by the subsequent depositions may also be responsible.

### 3.2. Electrochemical Impedance Spectroscopy

**Figure 8:**
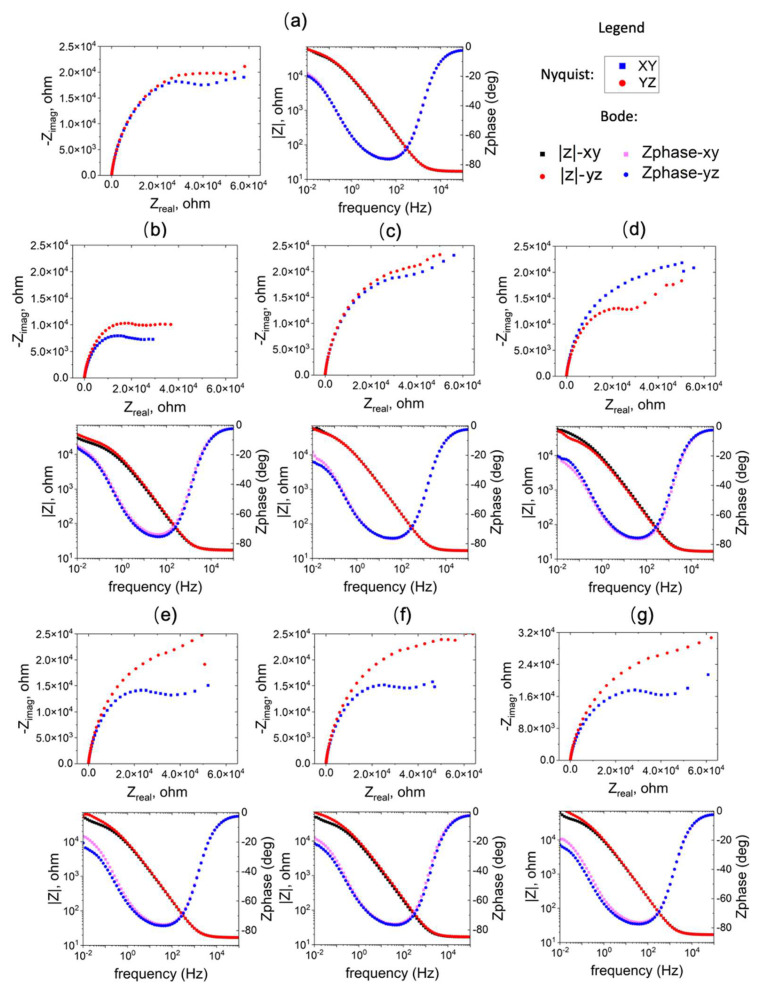
EIS results of the WAAM samples on the YZ plane; subfigures (**a**) corresponds to conventional sample, and (**b**–**g**) correspond to WAAM samples 1–6.

**Figure 9:**
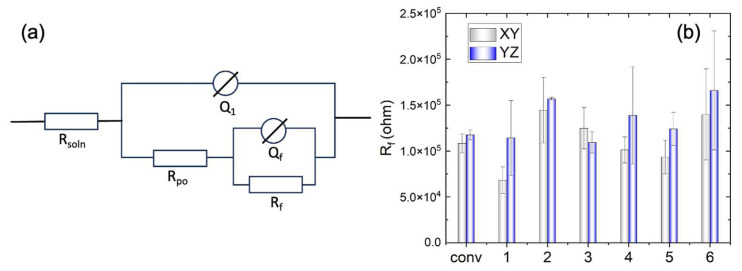
(**a**) Equivalent electrical circuit used to fit the impedance spectra. (**b**) The fitted parameter R_f_ of the conventional sample (conv) and WAAM samples under the different printing conditions; x-axis labels 1–6 indicate the WAAM samples 1–6.

To investigate the corrosion resistance of the WAAM Cu/Ni, impedance spectra were recorded at the OCP, repeating the process 3–4 times for each sample. Figure 8 displays both Bode and Nyquist plots for the selected tests. The bode plot illustrates a consistent trend in impedance frequency dependence across various conditions, with some discrepancies noted in the low-frequency domain (<1 Hz). In the Nyquist plot, the capacitive loop exhibits variable sizes based on processing conditions. Generally, a large semicircle suggests challenges in electron transfer between the substrate and solution. The high capacitance indicates a link between two processes: the formation of the passive oxide layer and the charging of the electric double layer. A secondary capacitive loop emerges at lower frequencies but displays some instability. The impedance spectra underwent analysis using the equivalent electrical circuit depicted in Figure 9a. Here R_soln_ is the electrolyte resistance, R_po_ is the resistance inside the film pores, Q_1_ the constant phase element (CPE) parallel to R_po_. R_f_ is the resistance of the corrosion product or film, and Q_f_ corresponds to the CPE parallel to R_f_. The use of a CPE is necessary due to a distribution of relaxation times resulting from heterogeneities at the electrode surface. The impedance of the CPE is given by

(1) $$Z_{CPE}=\frac{1}{Q}{\left(j\omega \right)}^{-n}$$

where Q represents the impedance of the CPE and *n* represents the empirical exponent, which can vary between 1 for a perfect capacitor and 0 for a perfect resistor. The important fitting parameters are listed in Table 3.

As can be seen in in Figure 9b, the film resistance (R_f_) of WAAM samples 2 and 6 surpasses that of the other conditions, exceeding that of the conventional sample. Conversely, sample 1 underperforms in comparison with the conventional ones. The elevated film resistance (R_f_) suggests a deceleration in the kinetics of the electrochemical process. This behavior may be attributed to a higher growth rate of the passive film or an increased thickness of the passive layer. Additionally, the resistance on the YZ plane is higher than that on the XY plane for the WAAM samples, except for sample 3, where the horizontal (XY) and vertical (YZ) planes exhibit similar resistance (R_f_).

Furthermore, the equivalent film thickness can be estimated from the fitted parameters derived from the EIS results with,

(2) $$d=\frac{\varepsilon \varepsilon_{0}A}{Q^{1/n}{\left(R_{f}\right)}^{(1-n)/n}}$$

where *d* is the film thickness; $\varepsilon_{0}$ is the vacuum permittivity (8.854 × 10^−12^ F m^−1^); ε is the dielectric constant, assumed to be 7.11 which is the value for cuprous oxide Cu_2_O; and *A* is the effective surface area of the sample. The derivation of Equation (2) is based on the equivalent film capacitance of the oxide layer that can be calculated with Equation (3), which is then related to the equivalent film thickness via Equation (4) [24,25],

(3) $$C_{eff}=Q^{1/n}{\left(R_{f}\right)}^{(1-n)/n}$$

(4) $$d=\frac{\varepsilon \varepsilon_{0}A}{C_{eff}}$$

The equivalent film thickness is thus calculated via Equation (2), and the values are listed in Table 3. The estimated surface film varies from tens to hundreds of nm.

### 3.3. ElectroPolarization Test

**Figure 10:**
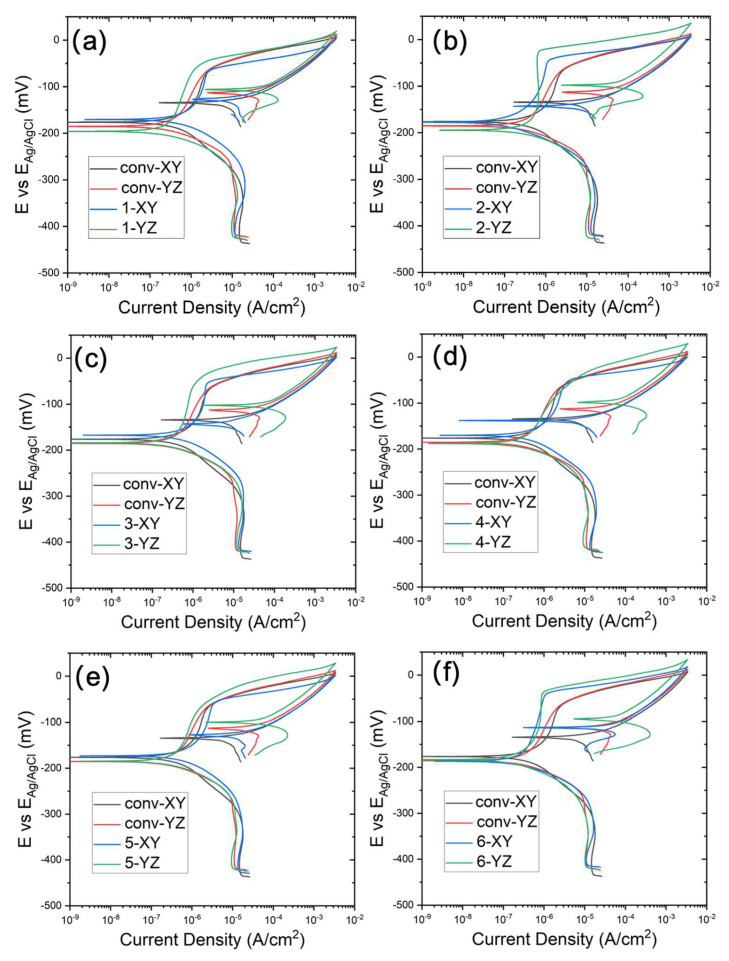
Potentiodynamic curve of WAAM samples: subfigures (**a**–**f**) correspond to WAAM samples 1–6; the conventional sample is presented in each subfigure as comparison.

**Figure 11:**
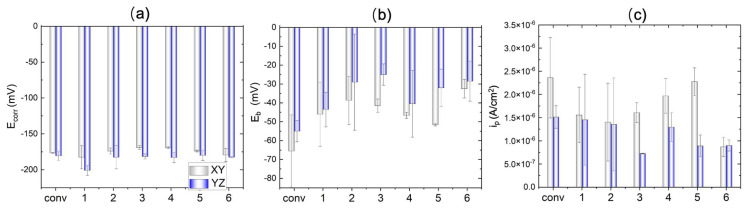
Extracted corrosion characteristics. (**a**) Corrosion potential, (**b**) breakdown potential, and (**c**) passive current density from the potentiodynamic test for conventional sample (conv) and WAAM samples under the different printing conditions; x-axis labels 1–6 indicate the WAAM samples 1–6.

The cyclic potentiodynamic polarization curves, tested in the 3.5 wt% NaCl solution, are displayed in Figure 10. The corrosion potential E_corr_ is summarized in Figure 11a, where the difference between the conventional and WAAM samples is quite small. The YZ plane has a slightly more positive E_corr_ than that of the XY plane. As the potential shifts further in the positive direction, the quick current density increase is associated with transpassive dissolution. The potential at the current density of 5 × 10^−5^ A cm^−2^ is measured as the breakdown potential E_b_ and is summarized in Figure 11b. Almost all of the WAAM samples outperform the conventional one. WAAM sample 6 has the highest overall E_b_ when considering both the XY and YZ planes. Moreover, the YZ plane has a higher E_b_ than that of the XY plane. The YZ plane for sample 3 has the highest E_b_.

The conventional samples (depicted in black and red lines) demonstrate a domain of low current density (<5 × 10^−5^ A cm^−2^) before the transpassive dissolution. This can be ascribed to the protective corrosion product film formed on the sample surface. The passive region is atypical, as the passive current density (i_p_) increases with a positively shifting applied potential. The passive current density over the potential window from −120 mV to −75 mV has been averaged and compared between the conventional and WAAM samples in Figure 11c. WAAM sample 6 exhibits the lowest passive current density among them and is significantly lower than that of the conventional sample. WAAM sample 2 also demonstrates a slightly lower current density than the conventional sample. Additionally, the passive current density is higher in the XY plane compared with that in the YZ plane. The YZ plane for sample 3 has the lowest i_p_.

## 4. Discussion

The film resistance (R_f_) measured by EIS, along with the E_b_ and i_p_ measured by cyclic polarization tests, reveals differences in corrosion resistance, passivity, and transpassive behaviors between the conventional and WAAM Cu/Ni samples. WAAM sample 2 (TS = 500 mm/min and track distance of 5.25 mm) and 6 (TS = 1000 mm/min and track distance of 3.85 mm) exhibit higher film resistance (R_f_) than the conventional sample in both XY and YZ build planes. Sample 6 also exhibits the lowest passive current density (i_p_) among the WAAM samples, significantly lower than that of the conventional sample. WAAM sample 2 also demonstrates a slightly lower current density than the conventional sample. In terms of breakdown potential E_b_, almost all of the WAAM samples outperform the conventional sample. WAAM sample 6 has the highest overall E_b_. Overall, the electrochemical analysis indicates that WAAM sample 6 has the best corrosion performance in the 3.5% NaCl solution. Moreover, the YZ plane exhibits higher film resistance at OCP, higher transpassive potential, and lower passive current density when compared with the XY plane, indicating better corrosion resistance.

**Figure 12:**
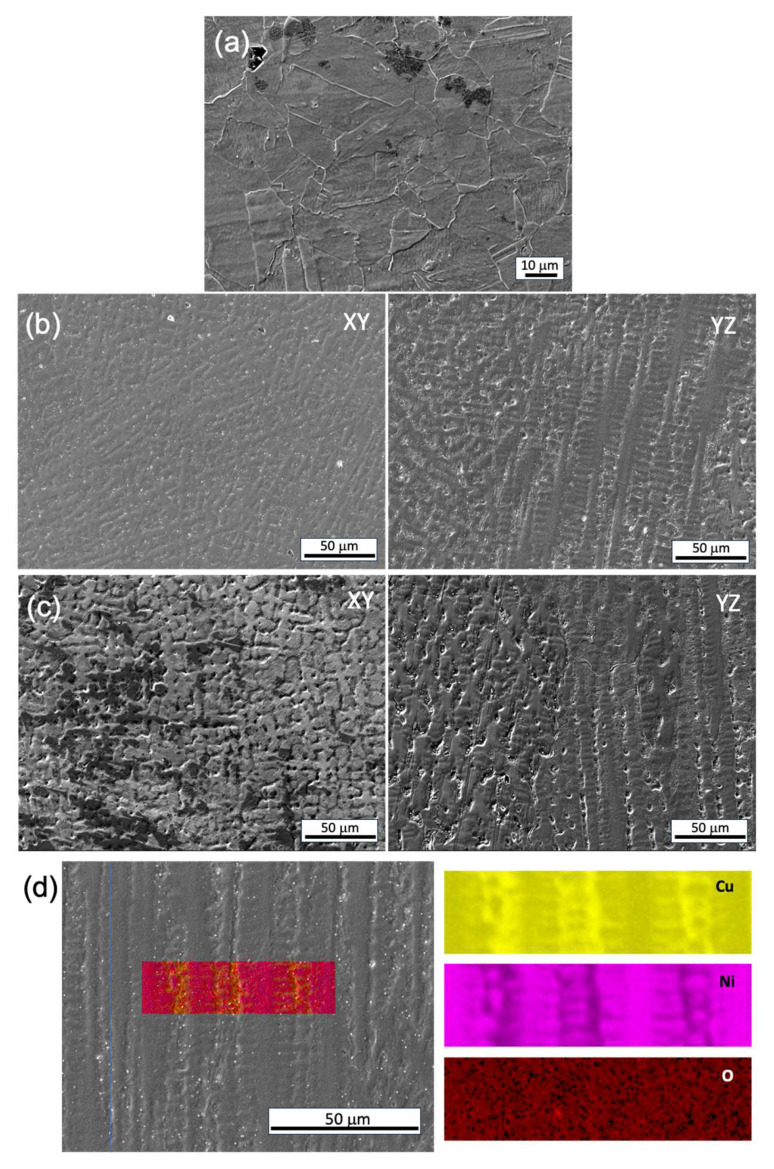
SEM image of the corrosion morphology post-potentiodynamic test. (**a**) Conventional sample, (**b**) WAAM sample 4, (**c**) WAAM sample 6, and (**d**) EDS area scan of the WAAM sample 6. The yellow, pink and red represents the concentration of Cu, Ni and O element, respectively; the brighter color indicates higher concentrations and the darker indicates lower concentrations.

**Figure 13:**
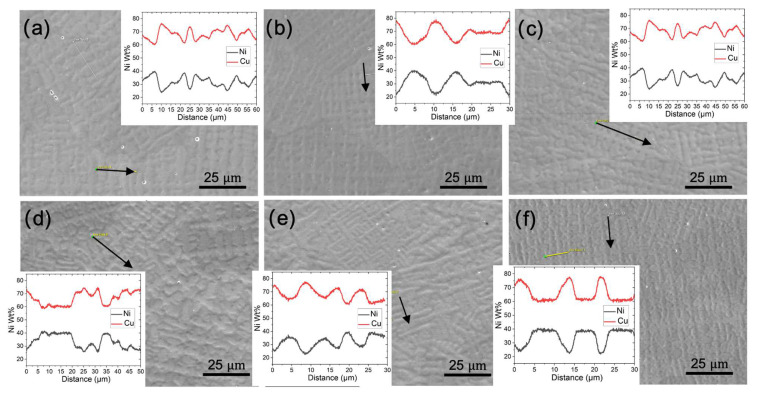
SEM images of the dendritic features on the XY plane, the arrow represents the EDS line scan and the results are shown in the subfigure inset. Subfigures (**a**–**f**) correspond to WAAM samples 1–6. A line scan was also taken along the thin yellow lines, and similar results were found as with the black ones, so they are not plotted again.

The corrosion morphologies of both the conventional and WAAM samples demonstrate uniform dissolution on the millimeter scale, but noteworthy differences in grain/subgrain level corrosion behavior exist. In the conventional sample, uneven corrosion depth is observed for grains with different orientations (Figure 12a). In the WAAM sample, the inter-dendritic area is less resistant to corrosion attack compared with the dendritic core area (Figure 12b,c), closely related to the uneven Ni element distribution revealed in Figure 12d. A quantitative analysis using EDS line scan, as seen in Figure 13, further reveals that the concentration difference between the dendritic core and inter-dendritic area can be as high as 20%. Due to the highly non-uniform distribution of the Ni element at different locations, even within the same sample, making comparisons among different printing conditions is not straightforward (Figure 13). The uneven Ni solute distribution could be ascribed to various factors, as follows. Firstly, the melt pool experiences a non-uniform cooling rate and temperature gradient. Typically, the bottom region undergoes a more rapid cooling rate and higher thermal gradient compared with the central molten pool. Secondly, dendrite growth direction plays a crucial role in solute diffusion. Dendrites growing along the heat flux direction allow solute diffusion into the liquid away from the interface. Conversely, dendrites growing obliquely could be hindered by preceding dendrites. Furthermore, the solute concentration ahead of inclined dendrite tips surpasses that of dendrites perpendicular to the molten pool’s isotherm [26]. Finally, subsequent deposition leads to repetitive thermal cycling, potentially causing grain coarsening and migration of Ni solute driven by concentration gradients. However, due to Ni’s slightly larger atomic radius compared with copper, the migration rate of atoms may become more challenging as the Ni content in the area increases. The increase of the Ni element enhances the corrosion resistance, as the corrosion rate of Ni is at least two orders of magnitude lower than Cu [27]. Ni could reside at the interface between the substrate/film interface or the inner side of the passive film [28]. This Ni enrichment could occur quickly in the early stage when exposed to seawater [28]. The contrast in corrosion resistance between the inter-dendritic and dendritic core areas is thus likely attributed to the depletion of Ni in the inter-dendritic area (Figure 12).

It has been reported that the passive film formed on Cu–Ni alloy is different from that on stainless steels, which is typically nanometer thick. The film on Cu–Ni takes a long time (several days to 2–3 months) at 15–17 °C, with a thickness in the micrometers [28]. Additionally, its protection of the matrix is not as desirable when compared with that of stainless steels [28]. The cuprous oxide (Cu_2_O) film produced is believed to be predominately responsible for the corrosion protection, and the thicker passive film mainly reduces the corrosion rate by inhibiting the cathodic processes, such as the oxygen reduction reaction on the sample surface [29]. In this work, the passive film formed during polarization test and that generated at OCP during the EIS test are formed within hours and may not be thick enough to inhibit the cathodic reaction by mitigating oxygen migration. However, it is still possible that the protective passive film is established within a few hours, as the associated discharge of copper ions can reduce 10-fold over 10 min and 100 fold within the first hour [30]. The estimated passive films from the EIS test listed in Table 2 vary from tens to hundreds of nanometers. There could be inaccuracies in this estimate, that arise from (1) the selection of the equivalent electric circuit and fitting process, (2) the assumption that the passive film is composed of cuprous oxide Cu_2_O because the products of corrosion reactions could form a multi-layered oxide structure [31]. This thickness range is much smaller than the film formed for a Cu–Ni alloy that is immersed in seawater for a much longer time [28]; however, the EIS test reveals a comparable film resistance to those exposed to much longer times [29], indicating that the film that forms on 70/30 Cu–Ni within hours can possess substantial protectiveness.

The difference in alloy elements (such as Ti and Si) between the conventional and WAAM Cu–Ni could also affect their corrosion performance. It is worth mentioning that there is no segregation present in the conventional condition. Understanding the variation in different corrosion behaviors associated with the minor element is challenging, especially in combination with the drastically different grain structures and segregation characteristics between the conventional and WAAM samples. We plan to explore the alloying element effect in our future study, by analyzing the passive film and corrosion product characteristics for the conventional and selected WAAM samples with the highest performances.

## 5. Conclusions

In this study, 70/30 Cu–Ni has been successfully fabricated by WAAM at TSs varying between 500 and 800 mm/min. A conventional 70/30 Cu–Ni was also investigated for comparison purposes. The as-built microstructures of WAAM Cu–Ni were analyzed, considering the grain structure, chemical segregation, and other defects. Corrosion properties were examined in a 3.5 wt% NaCl solution. The main conclusions are drawn as follows:

1. The as-fabricated microstructures are characterized by columnar grains with prominent dendrites. The longitudinal direction of the columnar grain deviates from the build direction. Strong chemical segregation has been observed in the form of Ni element depletion in the inter-dendritic area.
2. With increasing TS, the aspect ratio of columnar grains increases, resulting in a reduced size of the XY cross-section and an increased length of the YZ cross-section. The grain size also decreases with increasing track distance. A close-to-〈001〉 texture has developed on 30% to 40% of the area on the XY plane. The grain size of the conventional sample is one order of magnitude smaller compared with that of the WAAM samples and exhibits random orientation.
3. The corrosion performance is evaluated in terms of film resistance at OCP, the passive current density and breakdown potential during polarization. Two WAAM samples, produced under a TS of 800 mm/min and track distance of 5.25 mm as well as a TS of 1000 mm/min and track distance of 3.85 mm, have the best corrosion performance in the 3.5% NaCl solution.
4. Anisotropy in the corrosion performance has also been observed. The YZ plane exhibits higher film resistance at OCP, a higher breakdown potential, and lower passive current density, compared with the XY plane, indicating better corrosion resistance.
5. At the grain/subgrain level, the conventional Cu–Ni demonstrates crystallography-dependent corrosion resistance, whereas the WAAM Cu–Ni exhibits preferential corrosion attack at the inter-dendritic area due to the uneven distribution of Ni element. The reason behind the apparent corrosion enhancement in the aforementioned WAAM Cu–Ni samples compared with its conventional counterpart needs further analysis, which will be carried out in our study in the near future.

This study aims to provide a microstructural and corrosion analysis of the WAAM Cu–Ni processed by various printing parameters. It paves the way to a further understanding of the applicability of WAAM to copper-based alloys and the optimization of their corrosion properties though tailored microstructures, which themselves will be achieved through the optimization of the printing parameters and post-processing methods.

## Figures and Tables

**Table 1:** Chemical concentration of the 70/30 Cu–Ni wire and plate in wt%.

	Cu	Ni	Mn	Pb	Fe	Zn	P	Ti	Si
Plate	68.046	30.46	0.73	0.009	0.56	0.149	0.001	-	-
Wire	67.5	30	0.7	0.003	0.55	-	0.006	0.25	0.1

**Table 2:** WAAM parameters used to print 70/30 Cu–Ni single tracks.

Specimen	WFS	TS	Track Distance	Interpass Temp (°C)
1	8 m/min	500 mm/min	3 mm	150~200
2			5.25 mm	
3		800 mm/min	2.4 mm	
4			4.2 mm	
5		1000 mm/min	2.2 mm	
6			3.85 mm	

**Table 3:** Summary of the essential fitting parameters for the EIS results.

Specimen	R_f_ × 10^5^ (Ω)		Q_f_ × 10^−5^ (F)		*n*_f_		d_f_ (μm)	
	XY	YZ	XY	YZ	XY	YZ	XY	YZ
Wrought	1.08	1.18	2.44	2.37	0.34	0.32	0.13	0.095
1	0.68	1.14	2.84	2.83	0.340	0.29	0.20	0.041
2	1.44	1.57	2.13	2.29	0.29	0.31	0.063	0.051
3	1.25	1.09	2.54	2.98	0.29	0.31	0.046	0.049
4	1.01	1.39	2.36	2.34	0.31	0.30	0.12	0.058
5	0.93	1.24	2.43	2.04	0.32	0.33	0.14	0.14
6	1.40	1.66	2.68	2.47	0.27	0.28	0.021	0.023

## Data Availability

The data presented in this study are available on request from the corresponding author.
